# Colorectal Cancer: Advances in Prevention and Early Detection

**DOI:** 10.1155/2015/518068

**Published:** 2015-10-07

**Authors:** Anne Miles, Fränzel van Duijnhoven, Amy McQueen, Raymond Oliphant

**Affiliations:** ^1^Department of Psychological Sciences, Birkbeck, University of London, Malet Street, London WC1E 7HX, UK; ^2^Division of Human Nutrition, Wageningen University, P.O. Box 8129, 6700 EV Wageningen, Netherlands; ^3^School of Medicine, Washington University in St. Louis, Campus Box 8005, 4523 Clayton Avenue, St. Louis, MO 63110, USA; ^4^West of Scotland Cancer Surveillance Unit, University of Glasgow, 1 Lilybank Gardens, Glasgow G12 8RZ, UK

Colorectal cancer (CRC) is currently the fourth leading cause of cancer death worldwide. While mortality rates are in decline in most westernised countries, global estimates predict that CRC incidence rates and the overall number of CRC-related deaths are set to rise by 77% and 80%, respectively, by 2030. The development of CRC is multifactorial, and risk factors include various lifestyle, genetic, and environmental factors. It has been estimated that at least half of CRC cases could be prevented by a reduction in known modifiable lifestyle-related risk factors. Further reductions in CRC incidence and mortality can be achieved through screening, but the uptake of screening varies across different sectors of the population. This special issue comprises articles highlighting issues in the prevention, early diagnosis, and treatment of CRC.

J. Sovich et al. provide a comprehensive review of the effectiveness and adoption of existing technologies for CRC screening. They review stool-based, endoscopic, radiological, and serum-based methods for screening, discussing both commonly used methods, such as gFOBT and colonoscopy, and newly emerging ones, such as faecal DNA testing, capsule endoscopy, and serum-based tests. Colonoscopy remains the gold standard, but the authors highlight improvements in the sensitivity of the newer stool-based tests (FIT and faecal DNA tests) and raise the important point that “the best test is often the one that patients will do.”

J. Krok-Schoen et al. report the results of a randomised trial aimed at increasing CRC screening among adults living in Ohio Appalachia, an area with higher than average rates of both CRC incidence and mortality. The intervention comprised both a media campaign (billboards, posters, and newspaper articles) and a clinic intervention (brochures and posters). Randomisation was performed at the county level, with counties stratified by the proportion of people with late-stage diagnosis. The communication-focused intervention was not effective in promoting the uptake of screening, and the authors suggest that while such strategies may increase knowledge and awareness of screening, additional interventions or resources may be needed to change behaviour.

B. White et al. not only compared multiple interventions to increase stool blood test uptake in a national screening program but also examined multiple moderators of intervention effects to assess* what* is needed by* whom*. Only the combined intervention group (endorsement flyer plus kit enhancement plus community advertisements) significantly increased screening rates compared with controls for all participants, whereas all other significant intervention effects were conditional on other factors. More resources may be needed to increase screening uptake among people not previously screened, aged 70 and older, and living in economically deprived areas. Future research could examine the mediators of intervention effects in these population subgroups to reveal the mechanisms by which the interventions work and how they might be further enhanced. For example, all the interventions tested involved health communication approaches; however, individuals living in more deprived areas may need increasingly personal and novel interventions to compete with the cognitive demands of scarcity (see [1]).

S. H. Lo et al. explored potential pathways of influence on CRC screening uptake in a national screening program to better understand the processes that may influence behavior. Specifically, they explored factors that may explain sociodemographic differences in screening behaviour. Their cross-sectional results support future research to confirm cognitive determinants such as knowledge, perceived barriers to screening, and social norms as mediators of the effects of sociodemographic predictors on screening uptake. Elucidating the mechanisms and interrelations between known determinants of screening behaviour will enable improved message development and intervention design to increase screening rates and decrease disparities across sociodemographic subgroups.

A. Anderson et al. investigated the awareness of lifestyle risk factors associated with CRC in patients who had been diagnosed with a colorectal adenoma through a colorectal screening programme in Scotland, UK. Their study shows that the knowledge of relevant CRC risk factors was low in people at increased risk of the disease. The authors suggest exploring opportunities within routine CRC screening settings to raise awareness about lifestyle and prevention and to provide further guidance and personalised support to enhance the translation of improved knowledge into effective behavioural changes to reduce CRC risk.

C. Mojica et al. present the results of a large population-based study examining the relationship between population characteristics, English proficiency, and the diagnosis of advanced CRC in California, USA. This cancer registry based study found that late-stage CRC diagnosis was higher in areas with a greater proportion of recent immigrants and those of limited financial means. However, it suggests that amongst Hispanic groups a lower proportion of English proficiency was associated with lower odds of advanced disease. This interesting original paper highlights the complexity of the relationship between the patient, socioeconomic and neighbourhood characteristics, and CRC risk.

L. A. Siminoff et al. examine the patient and medical correlates of a missed diagnostic opportunity (MDO) among patients diagnosed with CRC in Virginia and Ohio, USA. Patients had experienced symptoms prior to diagnosis and were not diagnosed through routine screening. This study involved the review of patient medical records and found that a third of patients with symptoms presumptive of CRC experienced a delay in the diagnostic process for CRC. An MDO was more likely to occur among patients under 50 years of age, women, and patients who had seen a greater number of physicians. In addition to reminders and educational interventions for patients and providers about CRC and screening options, future applications of electronic medical records may reduce MDOs through systematic symptom recording and analysis.

M. Hav et al. present a comprehensive review of the prognostic implications in the pathological assessment of rectal cancer after treatment with chemoradiotherapy. The review highlights many of the challenges encountered in the interpretation and prediction of outcome after surgical resection in patients who have already undergone neoadjuvant therapy. The authors emphasize the importance of careful specimen handling in addition to accurate microscopic pathological assessment to achieve optimal prognostication in this group of patients and highlight several key areas of note.

In conclusion, CRC remains a significant health problem across the world. This special issue highlights the contribution different disciplines can make towards tackling CRC, through developments in screening technologies and treatment and understanding and encouraging lifestyle changes and participation in screening, as well as determining those at risk from late-stage at diagnosis. Innovations in methods of diagnosis and treatment can make vital contributions to CRC incidence and mortality; however, more research is needed to reduce disparities in access to and uptake of the best screening, diagnostic, and treatment methods that are available.

*Anne Miles* *Fränzel van Duijnhoven* *Amy McQueen* *Raymond Oliphant*
